# Modulating skin colour: role of the thioredoxin and glutathione systems in regulating melanogenesis

**DOI:** 10.1042/BSR20210427

**Published:** 2021-05-04

**Authors:** Yaoying Lu, Kathryn F. Tonissen, Giovanna Di Trapani

**Affiliations:** 1 School of Environment and Science, Griffith University, Nathan, QLD 4111, Australia; 2 Griffith Institute for Drug Discovery, Griffith University, Nathan, QLD 4111, Australia

**Keywords:** antioxidants, glutathione, hyperpigmentation, melanogenesis, thioredoxin

## Abstract

Different skin colour among individuals is determined by the varying amount and types of melanin pigment. Melanin is produced in melanocytes, a type of dendritic cell located in the basal layer of the epidermis, through the process of melanogenesis. Melanogenesis consists of a series of biochemical and enzymatic reactions catalysed by tyrosinase and other tyrosinase-related proteins, leading to the formation of two types of melanin, eumelanin and pheomelanin. Melanogenesis can be regulated intrinsically by several signalling pathways, including the cyclic adenosine monophosphate (cAMP)/protein kinase A (PKA), stem cell factor (SCF)/c-kit and wingless-related integration site (Wnt)/β-catenin signalling pathways. Ultraviolet radiation (UVR) is the major extrinsic factor in the regulation of melanogenesis, through the generation of reactive oxygen species (ROS). Antioxidants or antioxidant systems, with the ability to scavenge ROS, may decrease melanogenesis. This review focuses on the two main cellular antioxidant systems, the thioredoxin (Trx) and glutathione (GSH) systems, and discusses their roles in melanogenesis. In the Trx system, high levels/activities of thioredoxin reductase (TrxR) are correlated with melanin formation. The GSH system is linked with regulating pheomelanin formation. Exogenous addition of GSH has been shown to act as a depigmenting agent, suggesting that other antioxidants may also have the potential to act as depigmenting agents for the treatment of human hyperpigmentation disorders.

## Introduction

The colour of the skin, as well as hair and eyes, is determined by different levels and types of melanin. Melanin is not only responsible for the appearance of the skin but also plays an important photoprotective role in human skin against harmful ultraviolet radiation (UVR) [1,2].

The melanin pigment is produced in melanocytes, a type of dendritic cell located in the basal layer of the epidermis, through the process of melanogenesis [3]. Melanosomes, subcellular lysosome-like organelles in melanocytes, synthesize two types of melanin, the brownish-black eumelanin and the reddish-yellow pheomelanin [4]. The first, and also the rate-limiting step, of melanogenesis for both eumelanin and pheomelanin synthesis is the oxidation of tyrosine to dopaquinone by a copper-containing enzyme called tyrosinase [5]. After melanin synthesis, the mature melanosomes are transported to the neighbouring keratinocytes, which are also located in the epidermis [1]. Melanogenesis is initiated and regulated by a number of signalling pathways and transcription factors. It can also be modulated by some extrinsic factors such as UVR.

The production of melanin in melanocytes induced by UVR generates reactive oxygen species (ROS), which contributes to oxidative damage of DNA [6]. The formation of ROS and oxidative damage and/or the repair of damage produces important signals that stimulate melanogenesis [7–10]. Cellular antioxidant systems, such as the thioredoxin (Trx) and glutathione (GSH) systems, function to reduce oxidative stress [11,12]. Therefore, the antioxidant defence systems play a vital role in maintaining an optimal redox balance in melanocytes by quenching ROS and protecting against oxidative stress, excessive melanogenesis and photo-damaged skin. This review provides an overview of melanin formation, the role of oxidative stress in regulating melanogenesis, the involvement of antioxidant systems in melanogenesis and the utility of antioxidants for skin lightening.

## Melanin and melanogenesis

**Figure 1:**
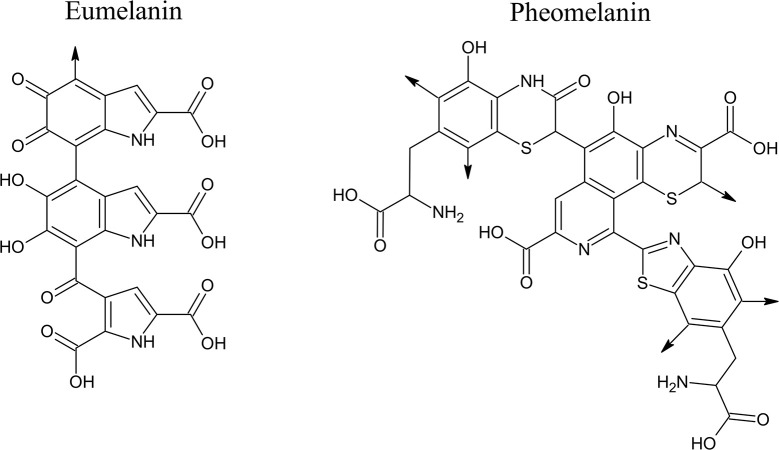
Chemical structures of eumelanin and pheomelanin The structure of eumelanin represents DHICA-melanin, which is a polymeric structure made of DHICA as the basic motif. The positions with –COOH group in the eumelanin structure can be substituted by –H to represent DHI-melanin. Both DHICA-melanin and DHI-melanin are regarded as eumelanin. The molecular weight of eumelanin shown above is 563.4 g/mol. The structure of pheomelanin consists of polymers composed of benzothiazine and benzothiazole units. The molecular weight of pheomelanin shown above is 806.8 g/mol. The arrows in the structures of eumelanin and pheomelanin indicate sites where polymerization can occur [15,16]. Abbreviations: DHI, 5,6-dihydroxyindole; DHICA, DHI-2-carboxylic acid. This figure is reproduced and used with permission from the publisher [15]. © 2007 The Authors. Journal Compilation. The American Society of Photobiology.

The skin colour is regulated by the production of varying levels of a dark biological pigment called melanin. There are two common types of melanin: the insoluble brownish-black eumelanin and the soluble reddish-yellow pheomelanin (Figure 1). Both eumelanin and pheomelanin are present in the human epidermis [4,13]. It was found that eumelanin is more abundant in darkly pigmented skin compared with light skin. Melanin is not only the primary determinant of skin colour but also protects the skin against the damaging effects of UVR [1,2]. Skin phototypes are categorized into six (I–VI) types by the Fitzpatrick skin type system based on the amount of melanin in the skin and their response to UVR exposure, where type I is very fair skin and always burns and type VI is black colour skin that never/rarely burns [14].

**Figure 2:**
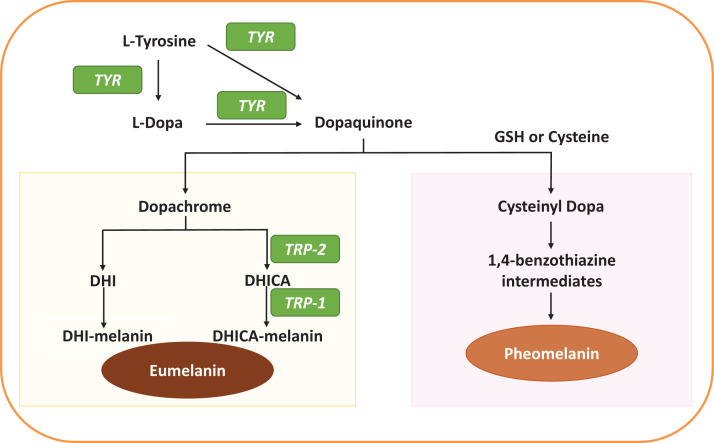
Melanogenesis formation pathway Eumelanin and pheomelanin are produced through a multistep process of biochemical reactions, with the rate-limiting enzyme TYR using l-tyrosine and/or l-dopa as the initial substrates (Abbreviations: DHICA, DHI-2-carboxylic acid; TRP-2, dopachrome tautomerase).

Melanogenesis is a complex multistep process characterized by a series of biochemical reactions (Figure 2). Tyrosinase (TYR), Tyrosinase-related proteins-1 (TRP-1) and TRP-2 (also known as dopachrome tautomerase) are the three important enzymes involved in melanogenesis. The first and also the rate-limiting step of melanogenesis for both eumelanin and pheomelanin is the oxidation of l-tyrosine and/or l-dopa to dopaquinone by a copper-containing enzyme called tyrosinase [5]. Dopaquinone can react with GSH or cysteine to form cysteinyl dopa that produces benzothiazine intermediates, which undergo oxidative polymerization for pheomelanin formation [17,18]. On the other hand, dopaquinone can also undergo intramolecular cyclization followed by an oxidation reaction to produce an orange intermediate, dopachrome [15]. Decarboxylation of dopachrome generates 5,6-dihydroxyindole (DHI), which rapidly oxidizes and polymerizes to form DHI-melanin. In the presence of TRP-2, dopachrome tautomerizes to yield DHI-2-carboxylic acid (DHICA), which then rapidly oxidizes and polymerizes to form DHICA-melanin by TRP-1 [19]. Both DHI-melanin and DHICA-melanin are regarded as eumelanin.

Since melanogenesis is a complex process, various pigmentary disorders occur when it goes awry. Pigmentary disorders are classified into hyperpigmentation, hypopigmentation and mixed pigmentation (hypopigmented and hyperpigmented). Hyperpigmentation is a dermatological condition that occurs when patches of skin are darker than the natural colour of the skin, due to excessive production of melanin, and can result in serious dermatological problems such as ephelides (freckles), solar lentigines, melasma and post-inflammatory hyperpigmentation. Hypopigmentation refers to any form of reduced pigmentation, the most common conditions include vitiligo, albinism and post-inflammatory hypopigmentation [20].

## Regulation of melanogenesis

### Intrinsic regulation of melanogenesis

**Figure 3:**
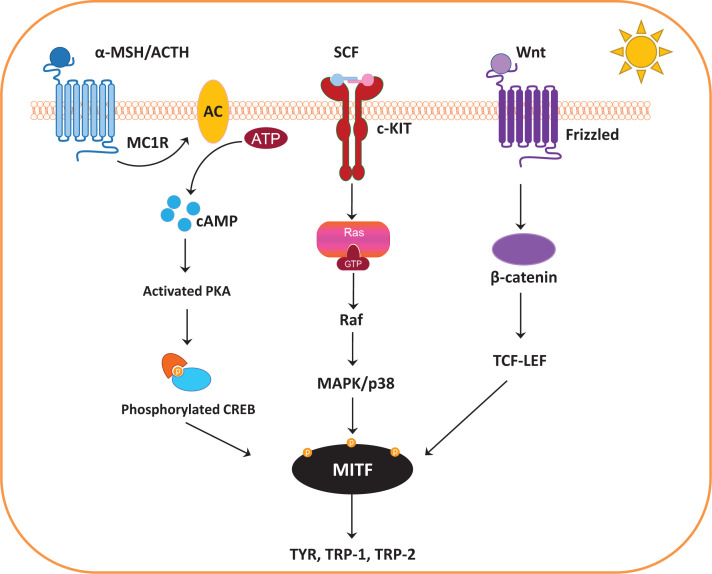
Melanogenesis signalling pathways The central melanogenesis regulator MITF can be regulated by different signalling pathways, including the cAMP/PKA signalling pathway, SCF/c-kit mediated signalling pathway and Wnt/β-catenin signalling pathway (Abbreviations: α-MSH, α-melanocyte-stimulating hormone; AC, adenylate cyclase; ACTH, adrenocorticotropic hormone; ATP, adenosine triphosphate; cKIT, tyrosine-protein kinase kit; CREB, cAMP response element-binding protein; GTP, guanosine-5′-triphosphate; MAPK, mitogen-activated protein kinase; MC1R, melanocortin 1 receptor; TCF-LEF, T-cell factor/lymphoid enhancer-binding factor; TRP-2, dopachrome tautomerase).

Skin melanogenesis is modulated by a number of signalling molecules and transcription factors. Melanogenesis-associated transcription factor (MITF) is the most important regulator of melanogenesis, regulating the expression of the melanogenesis-related proteins, such as TYR, TRP-1 and TRP-2 [21]. Moreover, MITF is involved in the regulation of other transcription factors, which control the differentiation [22], proliferation, survival [23] and motility [24] of melanocytes. MITF can be regulated by different signalling pathways, including the cyclic adenosine monophosphate (cAMP)/protein kinase A (PKA) signalling pathway [25], the stem cell factor (SCF)/c-kit mediated signalling pathway [26,27] and the wingless-related integration site (Wnt)/β-catenin signalling pathway [28]. These signalling pathways are activated by the binding of different ligands to their specific melanocyte cell surface receptors (Figure 3).

The cAMP/PKA signalling pathway is activated by the binding of α-melanocyte-stimulating hormone (α-MSH) or adrenocorticotropic hormone (ACTH) to the melanocortin 1 receptor (MC1R). MC1R is a member of the class A family of G protein-coupled receptors [29,30]. The two melanocortin peptides, α-MSH and ACTH, are the agonists of MC1R and are both derived from the same precursor, called pro-opiomelanocortin (POMC), by proteolytic cleavage [31]. Binding of α-MSH/ACTH to MC1R activates adenylate cyclase (AC), which catalyses the production of the secondary messenger cAMP [32]. Increasing levels of cAMP activate PKA, which phosphorylates cAMP response element-binding protein (CREB) for further downstream gene expression regulation. Phosphorylated CREBs then induce the expression of MITF, which stimulates the expression of TYR, TRP-1 and TRP-2 to allow melanin synthesis (Figure 3).

The Wnt/β-catenin signalling pathway also contributes to the expression of MITF. The central controller in this pathway is β-catenin, which is the target for the ubiquitin-dependent degradation via phosphorylation by glycogen synthase kinase-3β (GSK3β) [33]. Binding of Wnt to its receptor Frizzled causes the inactivation of GSK3β, leading to the stabilization and accumulation of cytoplasmic β-catenin [34]. Stabilized β-catenin translocates to nuclei and binds to T-cell factor/lymphoid enhancer-binding factor (TCF-LEF) to induce expression of the MITF gene [35,36].

The SCF/c-kit mediated signalling pathway is activated by the binding of the SCF to the receptor called tyrosine-protein kinase kit (c-kit). This process initiates the mitogen-activated protein kinase (MAPK) cascades for further downstream pathways that regulate melanogenesis. Three major MAPKs are c-JUN N-terminal kinase (JNK), p38 and extracellular signal-regulated kinase (ERK). The phosphorylation of p38 activates MITF expression, which up-regulates melanogenesis-related proteins and stimulates melanin synthesis [37,38]. In contrast, several studies suggested that the activation of ERK and JNK phosphorylates MITF at Ser^73^, which leads to its ubiquitination and degradation, thus subsequently down-regulating melanogenesis (not shown in Figure 3) [26,39–41].

### Extrinsic regulation of melanogenesis

Skin pigmentation can also be modulated by extrinsic factors including UVR. UVR can directly aggravate melanin production by inducing the proliferation and activation of melanocytes and increasing the number of melanosomes in each melanocyte [42,43] as well as the rate of transfer of melanosomes from melanocytes to keratinocytes [44]. On the other hand, UVR stimulates melanocytes to secrete signal molecules such as α-MSH, ACTH or other bioactive peptides, inducing the melanogenesis signalling pathway for melanin synthesis [45,46]. As a result, the main melanogenesis regulator MITF is elevated after UVR, followed by an increase in other melanosomal proteins such as TYR, TRP-1 and TRP-2, leading to the stimulation of melanogenesis [47].

## ROS and melanogenesis

### ROS

ROS are chemical reactive species derived from oxygen and include hydrogen peroxide (H_2_O_2_), superoxide (O_2_^•−^) and the hydroxyl radical (OH•) [48]. It is known that the ROS produced in the skin by UVR are the key contributors to oxidative damage to the skin [49]. Excessive production of ROS causes oxidative stress, resulting in abnormal cellular and physiological functions, causing damage to DNA [50], proteins [51] and lipids [52]. ROS also regulate the synthesis of melanin through a number of mechanisms, as discussed further below.

### ROS and melanogenesis

Melanogenesis is an oxygen-dependent process, which consists of a sequence of oxidation reactions accompanied by the generation of O_2_^•−^ and H_2_O_2_. The catalytic activity of tyrosinase in the oxidation of l-Dopa to dopaquinone results in the production of O_2_^•−^ [53]. During the final stages of eumelanogenesis, H_2_O_2_ generation was associated with the oxidation of the eumelanin precursors DHI and DHICA [54]. Also, elevated levels of ROS and decreased concentrations of intracellular GSH were detected during melanogenesis [55]. This makes melanogenesis a potential source of ROS in pigmented cells, which in turn subjects melanocytes to high levels of oxidative stress. On the other hand, ROS were reported to stimulate/regulate melanogenesis. Low levels of H_2_O_2_ (≤0.3 mM) were found to activate tyrosinase, followed by increased melanin synthesis in human melanocytes [8]. In B16 mouse melanoma cells, O_2_^•−^ was demonstrated to mediate short-term UVR-induced melanogenesis, and this was not associated with the activation of tyrosinase [9]. UVA-mediated ROS production was reported to be the critical cellular signal for melanin production through the intricate interplay with cytosolic and mitochondrial Ca^2+^ in human melanocytes [10]. Since ROS are considered to play important roles in melanogenesis it stands to reason that, scavengers of ROS, such as antioxidants or antioxidant systems, may reduce hyperpigmentation or decrease melanogenesis.

## Antioxidant systems and melanogenesis

The antioxidant defence systems play a vital role in maintaining an optimal redox balance in melanocytes by quenching ROS, protecting against oxidative stress, excessive melanogenesis and photo-damaged skin. The endogenous antioxidants in melanocytes comprise enzymes, such as superoxide dismutase (SOD), catalase (CAT), glutathione peroxidase (GPx), glutathione reductase (GR) and Trx reductase (TrxR). Non-enzymatic antioxidants include GSH, ascorbic acid (Vitamin C) and α-tocopherol (Vitamin E) [56].

It was reported that CAT is a major enzymatic antioxidant in melanocytes, and it scavenges H_2_O_2_, a by-product of the melanogenic process. A decrease in CAT activity makes lightly pigmented cells less efficient in scavenging ROS and therefore cells are more susceptible to UV damage [57]. SOD, CAT and GPx are the first-line defence antioxidant enzymes [58], and the alterations of their activities have consequences for melanogenesis [59–61]. Vitamin C is widely used as a whitening agent because of its inhibitory effect on melanogenesis. The combined use of Vitamins C and E was also shown to be more effective for the treatment of hyperpigmentation than either vitamin C or E alone [62]. Quevedo also reported that UVR-induced proliferation and melanogenesis of melanocytes were reduced by topical applications of vitamins C and E to the skin of hairless mice [63]. The main cellular antioxidant systems, the Trx and GSH systems, also have reported roles in melanogenesis, which are the main focus of this review.

### The Trx system

The Trx system was first found in *Escherichia coli* [11]. It is one of the major antioxidant systems that can function against oxidative stress and it is present in all species [64,65]. It is composed of Trx, TrxR and nicotinamide adenine dinucleotide phosphate (NADPH) [11]. The Trx system functions through thiol-disulphide interchange reactions, contributing to many essential cellular functions, such as gene expression [66], DNA synthesis [67], signal transduction [68], cell growth and apoptosis [69].

**Figure 4:**
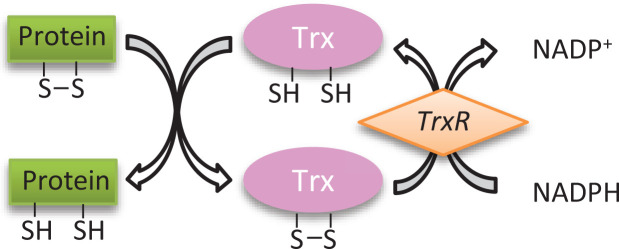
Mechanism of action of the Trx system The active form of Trx has two free thiol groups to catalyse target protein substrate reduction during which a disulphide bond is formed between two cysteine residues. TrxR catalyses the reduction of oxidized Trx using NADPH.

Trx is expressed as three different isoforms: Trx1, Trx2 and SpTrx in mammals, and they are located in the cytoplasm, mitochondria and spermatozoa respectively [70]. This review focuses on cytoplasmic Trx1. Trx1 is a small protein (approx. 12 kDa), which is encoded by the *TXN* gene and functions as a redox protein in many organisms [64]. The highly conserved active site (–Cys–Gly–Pro–Cys–), and the two cysteine residues at positions 32 and 35 are responsible for the disulphide reductase activity of Trx1 by reducing disulphide bonds on target proteins to form free thiol groups [11] (Figure 4). The thiol groups of the active site cysteine residues of Trx1 are oxidized to form a disulphide bond [71]. To maintain the appropriate level of reduced Trx1, oxidized Trx1 is reduced by an enzyme called Trx reductase 1 (TrxR1) and the reducing agent NADPH [11] (Figure 4).

### The Trx system and melanogenesis

It has been shown that reduced Trx suppresses melanin synthesis by reacting with the binuclear copper centre of tyrosinase, thus inhibiting tyrosinase activity [72]. TrxR, another component of the Trx system, has also been reported to be involved in melanogenesis. Schallreuter and colleagues found that TrxR levels were induced by X-ray related oxidative stress in response to the presence of ROS in the epidermis. This induction of TrxR was concomitant with melanin production in guinea pig epidermis and Cloudman S91 melanoma cells [73]. It was also reported that TrxR activity correlates with different skin phototypes I–VI (Fitzpatrick skin type system), where darker skin has significantly higher enzyme activity compared with very fair skin [74,75]. In addition, stable knockdown of TrxR1 resulted in a significant decrease in melanin levels and tyrosinase activity in melanocytes [76]. These studies suggested that high levels/activities of TrxR were correlated with melanin formation, providing additional information on the roles of cellular antioxidant proteins in melanogenesis (Table 1).

**Table 1:** The Trx and GSH systems in melanogenesis

Cellular antioxidants	Role in the antioxidant systems	Role in melanogenesis	References
**Trx (Thioredoxin)**	Acts as an antioxidant by facilitating the reduction in other proteins through thiol-disulphide interchange reactions	Suppresses melanin synthesis by reacting with the binuclear copper centre of tyrosinase	[11,72]
**TrxR (Thioredoxin reductase)**	Catalyses the reduction of oxidized Trx using NADPH	High levels/activities of TrxR were correlated with melanin formation	[11,73–75]
**GSH (Glutathione)**	One of the most abundant thiol antioxidants in cells that protects cells against oxidative stress	Involved in pheomelanin formation	[82–84]
**xCT (Cystine/glutamate antiporter)**	Provides intracellular cystine for GSH production	Acts as a major regulator of the production of pheomelanin by providing cystine	[90]
**GCL (Glutamate cysteine ligase)**	Catalyses the first step in the production of GSH	Increased GCL levels were related to the inhibition of UVA-induced melanogenesis	[93–95]
**GR (Glutathione reductase)**	Catalyses the reduction of oxidized glutathione (GSSG) to reduced glutathione (GSH)	High GR activity was associated with pheomelanin formation	[99]

### The glutathione system

**Figure 5:**
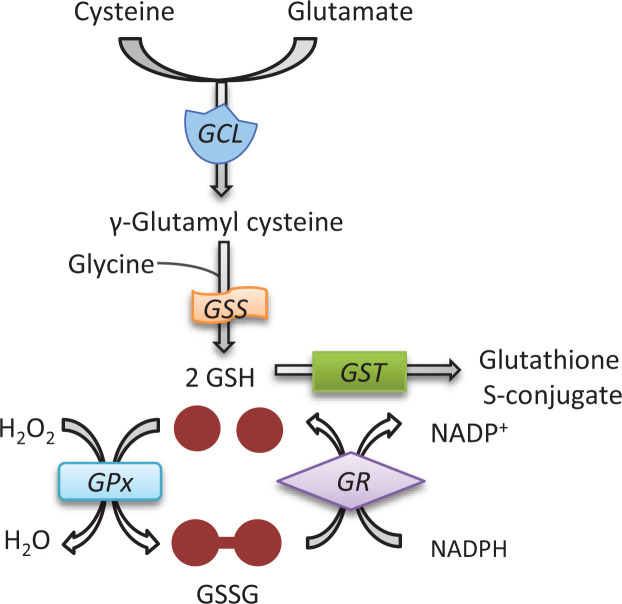
The glutathione system First, γ-glutamyl cysteine is synthesized from l-glutamate and cysteine using GCL. Then, GSS catalyses the condensation of γ-glutamyl cysteine and glycine to form GSH. GSH is used as a cofactor by GPx to reduce H_2_O_2_, resulting in the formation of GSSG. GR reduces GSSG to two GSH by NADPH (Abbreviation: GST, glutathione S-transferase).

To date, most attention regarding the role of antioxidant systems in melanogenesis has focused on the glutathione system. The glutathione antioxidant system consists of GSH, glutathione disulphide (GSSG), glutamate cysteine ligase (GCL), glutathione synthetase (GSS), GPx, GR and NADPH (Figure 5). GSH is synthesized in two consecutive steps which are catalysed by GCL and GSS respectively. During the first step that is considered rate limiting [77], γ-glutamyl cysteine is produced from l-glutamate and cysteine in the presence of GCL. The second step is catalysed by GSS and involves the condensation of γ-glutamyl cysteine and glycine to form GSH. GSH is a tripeptide (γ-Glu–Cys–Gly) and can exist in either a reduced (GSH) or an oxidized form (GSSG) [78,79]. GPx utilizes GSH as a cofactor to reduce H_2_O_2_, resulting in the formation of GSSG, which in turn can be reduced to GSH by GR using the electron donor NADPH [80,81]. GSH plays an important role in cellular processes such as cell proliferation and apoptosis, and in decreasing oxidative stress caused by ROS. Therefore, maintaining GSH in a reduced state is very important for optimal cell function.

### The glutathione system and melanogenesis

#### Glutathione and melanogenesis

It has been shown that GSH not only acts as an antioxidant by scavenging free radicals but is also involved in pheomelanin formation (Figure 2). The involvement of GSH in melanogenesis is mediated by two different mechanisms. First, the direct interaction between GSH and the active site of tyrosinase, resulting in the activation of tyrosinase activity when the concentrations of GSH is below 3 mM and inhibition at higher GSH concentrations [82]. Second, the reaction of the GSH thiol group with dopaquinone, leading to the formation of a sulphydryl–dopa conjugate and finally to a sulfur-containing pigment, pheomelanin instead of eumelanin [82–84]. Conversely, del Marmol and colleagues showed that the inhibition of GSH synthesis promoted tyrosinase activity and favoured eumelanogenesis in human melanoma cells [85]. Therefore, GSH, as an antioxidant with anti-melanogenic properties, has recently become the focus of attention in evaluating its suitability as a skin whitening agent.

Oral administration of GSH resulted in the lightening of skin colour in humans who were orally administered 500 mg of GSH per day for 4 weeks compared with the placebo group [86]. Similarly, Handog and colleagues reported a significant reduction in melanin index at both sun-exposed and sun-protected skin areas in all the subjects who received a GSH-containing lozenge (500 mg) daily for 8 weeks [87]. In 2018, a clinical trial (NCT04105504) in Indonesia also demonstrated that consumption of an oral GSH capsule (500 mg) once a day for 12 weeks reduced spot UV, spot polarization and skin tone measured at five different sites. Moreover, the oxidized form of glutathione (GSSG) was also found to have anti-melanogenic effects in humans. The skin melanin index was significantly lowered when 2% (w/w) GSSG lotion was applied on the face for 10 weeks compared with treatment with a placebo [88]. In addition, people who orally took GSSG (250 mg/day) for 12 weeks were found to have a lower melanin index and fewer UV spots at all sites, including the face and arm, compared with the placebo group [89]. These studies supported the potential of GSH to act as a skin whitening agent, however, the long-term efficacy and safety of GSH for skin lightening or the treatment of hyperpigmentation remains unclear.

#### Glutathione-related enzyme and melanogenesis

Modulation of the glutathione system through its key enzymes, including cystine/glutamate antiporter (xCT), GCL and GR, has also been correlated with melanin formation.

The xCT provides intracellular cystine, which is rapidly reduced to cysteine for GSH production. GSH/Cysteine then switches eumelanin synthesis to pheomelanin by conjugating with dopaquinone to become intermediates of pheomelanin (Figure 2). xCT was reported as a major regulator of the production of pheomelanin in hair and melanocytes, with minimal or no effects on eumelanin [90]. Overexpressed xCT was responsible for the reduction in eumelanin formation in melanocytes and increased production of pheomelanin in the sheep wool, where brown/yellow patches were observed [91]. In addition, it was reported that up-regulation of xCT through the mTOR pathway enhanced pheomelanin production and inhibited eumelanin formation, which altered the overall melanin synthesis in mice [92]. These studies provided information regarding the role that xCT plays in melanogenesis and suggest that further exploration should be undertaken to investigate its role in skin pigmentation.

GCL is the first enzyme of the cellular GSH biosynthetic pathway and it is composed of a catalytic subunit (GCLC) and a modifier subunit (GCLM). The up-regulation of GCL was related to the inhibition of UVA-induced melanogenesis. A Thai herbal formula (AVS073 formula) was shown to have inhibitory effects on UVA-induced melanogenesis, and this was associated with a redox mechanism involving up-regulation of GSH biosynthesis caused by the increased mRNA expression of GCLC and GCLM in human melanoma cells [93]. Similarly, the anti-melanogenic effect of dietary phenolics was also related to their antioxidant properties. The anti-melanogenic effect of gallic acid correlated with increased GSH levels as well as with up-regulated GCL mRNA expression in both G361 and B16F10 melanoma cells [94]. Other dietary phenolics, including caffeic acid, quercetin and avobenzone, protect against UVA-induced melanogenesis through up-regulation of Nrf2 activity and consequently increased expression of its target genes and proteins including GCLC in B16F10 cells [95].

GR is the enzyme responsible for maintaining reduced GSH by catalysing the reduction of GSSG to GSH. It was reported that GR plays an important role in the regulation of the types of melanin pigments formed, where high GR activity was associated with pheomelanin formation [96,97]. This may be due to the supply of reduced GSH by GR leading to pheomelanin formation. Ito proposed that melanogenic switching between eumelanin and pheomelanin production may be due to the covalent binding of dopaquinone to GR [98]. Also, melanogenic switching was found to be related to tyrosinase activity levels, where high tyrosinase activity produced an excess of dopaquinone resulting in the inactivation of GR and other enzymes essential for pheomelanogenesis [99]. These studies support the involvement of the GSH system in melanogenesis, and further investigation is needed to explore the possibility of utilizing, or targeting, glutathione system related enzymes in manipulating skin pigmentation.

A summary of the roles of the Trx and glutathione systems in melanogenesis is provided in Table 1.

## Current status and future directions

Oxidative stress induced by exogenous factors such as UVR contributes to the stimulation of melanogenesis. Antioxidants that function to reduce oxidative stress may demonstrate some efficacy in down-regulating excessive melanogenesis. GSH, one of the most abundant antioxidants in cells, has recently gained interest in its potential to act as a skin whitening agent. However, the long-term whitening effect of GSH remains unclear. We have reviewed the mechanism of the Trx and glutathione antioxidant systems, and the roles of these two antioxidant systems in melanogenesis, suggesting that antioxidants have the potential to act as depigmenting agents for the treatment of human hyperpigmentation disorders. Further investigation should focus on enhancing the stability and efficacy of topical or oral antioxidant products against hyperpigmentation for long-term use.

## Open Access

Open access for this article was enabled by the participation of Griffith University in an all-inclusive *Read & Publish* pilot with Portland Press and the Biochemical Society under a transformative agreement with CAUL.

## Abbreviations

α-MSH: α-melanocyte-stimulating hormone
ACTH: adrenocorticotropic hormone
cAMP: cyclic adenosine monophosphate
CAT: catalase
CREB: cAMP response element-binding protein
DHI: 5,6-dihydroxyindole
DHICA: DHI-2- carboxylic acid
ERK: extracellular signal-regulated kinase
GCL: glutamate cysteine ligase
GCLC: GCL catalytic subunit
GCLM: GCL modifier subunit
GPx: glutathione peroxidase
GR: glutathione reductase
GSH: glutathione
GSS: glutathione synthetase
GSSG: glutathione disulphide
GST: glutathione S-transferase
H_2_O_2_: hydrogen peroxide
JNK: c-JUN N-terminal kinase
L-DOPA: L-3,4-dihydroxyphenylalanine
MAPK: mitogen-activated protein kinase
MC1R: melanocortin 1 receptor
MITF: melanogenesis-associated transcription factor
NADPH: nicotinamide adenine dinucleotide phosphate
Nrf2: nuclear factor erythroid 2–related factor 2
O_2_^•−^: superoxide
OH•: hydroxyl radical
PKA: protein kinase A
POMC: pro-opiomelanocortin
ROS: reactive oxygen species
SCF: stem cell factor
SOD: superoxide dismutase
TCF-LEF: T-cell factor/lymphoid enhancer-binding factor
TRP-1: tyrosinase-related protein 1
TRP-2: dopachrome tautomerase
Trx: thioredoxin
TrxR: Trx reductase
TYR: tyrosinase
UVR: ultraviolet radiation
Wnt: wingless-related integration site
xCT: cystine/glutamate antiporter
